# Simulation and Study of Influencing Factors on the Solidification Microstructure of Hazelett Continuous Casting Slabs Using CAFE Model

**DOI:** 10.3390/ma17081869

**Published:** 2024-04-18

**Authors:** Qiuhong Pan, Wei Jin, Shouzhi Huang, Yufeng Guo, Mingyuan Jiang, Xuan Li

**Affiliations:** 1 School of Materials Science and Engineering, Jiangsu University, Zhenjiang 212013, China; 18852890810@163.com (W.J.); 13844075309@163.com (Y.G.); s1245350421@163.com (M.J.); lx595962@163.com (X.L.); 2 Inner Mongolia Liansheng New Energy Materials Co., Ltd., Tongliao 029200, China; shouzhi.huang@dingshenggroup.com

**Keywords:** Hazelett continuous casting, numerical simulation, CAFE, equiaxed crystal

## Abstract

The Hazelett continuous casting and rolling process represents a leading-edge production method for cold-rolled aluminum sheet and strip billets in the world. Its solidification microstructure significantly influences the quality of billets produced for cold rolling of aluminum sheets and strips. In this study, employing the CAFE (Cellular Automaton—Finite Element) method, we developed a coupled computational model to simulate the solidification microstructure in the Hazelett continuous casting process. We investigated the impact of nucleation parameters, casting temperature, and continuous casting speed on the microstructural evolution of the continuous casting billet. Through integrated metallographic analyses, we aimed to elucidate the controlling mechanisms underlying the Hazelett continuous casting process and its resultant microstructure. The results demonstrate that the equiaxed rate of grains increases with an increase in nucleation density, and the grain size decreases under constant cooling strength. With other nucleation parameters held constant, the grain size decreases as undercooling increases, and the columnar crystal zone expands. The nucleation density of the Hazelett continuous casting aluminum alloy has been determined to range between 10^11^ m^−3^ and 10^13^ m^−3^, and the undercooling ranges between 1 °C and 2.5 °C. The solidified grain structure can be controlled between 35 μm and 72 μm. The grain size of the continuous casting billet increases with an increase in pouring temperature and decreases as the casting speed increases. Elevating the pouring temperature positively impacts the fraction of high-angle grain boundaries and promotes the dendritic to equiaxed grain transition. Moreover, there exists potential for further optimization of continuous casting process parameters.

## 1. Introduction

Hazelett continuous casting and rolling has emerged as a pre-eminent production method for cold-rolled aluminum strip billets due to its streamlined process, reduced energy usage, cost-effectiveness, and enhanced efficiency [1]. In the production cycle, the key technical attributes of the Hazelett casting apparatus guarantee a nearly hermetic transition from liquid aluminum to the casting billet. The complexity of double-band solidification heat transfer and the variability in the thermal field in aluminum melt flow under liquid–solid coupling directly affect the characteristics of the solidification structure. Such complexity hampers the control over the microstructure and properties of subsequent multi-stand hot continuous rolling billets. The resulting billet defects are not easily ameliorated in the downstream cold rolling and foil rolling processes [2], representing one of the bottlenecks that restrict the production of high-precision, high-performance, and superior-quality strips and foils by continuous casting and rolling slabs. A wide array of enterprises and scholars have engaged in comprehensive research on the continuous casting solidification process. Tian et al. [3] undertook comprehensive studies on how variations in superheat affect the Columnar to Equiaxed Transition (CET) in solidification structure and grain size throughout the continuous casting process. Li et al. [4] developed a numerical model for calculating the pulse electromagnetic field, flow field, and temperature field in continuous casting, and conducted coupling simulations of multiple physical fields under different casting parameters and pulse magnetic oscillation parameters. The observations revealed that higher peak currents and casting speeds notably enhance the proportion of equiaxed crystal zones, attributable to Pulse Magnetic Oscillation (PMO) effects. Liu et al. [5] developed the SOLA model for calculating the flow field and implemented the Volume of Fluid (VOF) model for determining the fluid volume distribution. Numerical simulations of the flow field during the rapid solidification process of an aluminum strip double strip yielded the velocity field and images of the rapid solidification process of molten metal. The investigation encompassed the impact of cooling zone velocity and pouring temperature on flow dynamics. Chen [6] developed a two-dimensional continuous casting zone model to enhance heat transfer conditions between cooling water and aluminum melt. By modifying the Hazelett continuous casting process parameters, researchers determined the effects of aluminum melt inlet temperature, continuous casting velocity, and coating material on the continuous casting temperature field. Pavan Kumar Penumakala [7] created a finite element model to map the temperature evolution process, including the growth curve of the slab and the length of the solidification zone in the double-band continuous casting solidification ingot. Utilizing the moving slice model and integrating actual continuous casting industrial data, the study analyzed the influence of process parameters on the temperature and solidification fields in detail. Noii et al. [8] proposed a problem of seven different boundary values in coupled multi-field systems using Bayesian inversion technology, providing a reliable data calibration technique for computational mechanics models. Khodadadian et al. [9] proposed a parameter estimation framework in the variational phase field fracture problem, and used Bayesian inversion method to solve the problem of time and grid dependence. This framework was subsequently validated, yielding a load–displacement curve congruent with the established reference. Hu et al. [10] developed a two-dimensional transient coupled finite element model for calculating the temperature and stress fields inside the billet, analyzing heat transfer during solidification, examining cooling conditions, and optimizing process parameters. The aforementioned scholars have established a solid research foundation for modeling the flow thermal field and solidification field length in Hazelett double-belt continuous casting.

Despite the significance of microstructure dendrite formation, transformation, and influencing factors in Hazelett continuous casting, research in this area remains scarce. Since the 1990s, Rappaz and Gandin [11] have proposed the cellular automata (CA) method to simulate the microstructure, successfully predicting the transformation from columnar to equiaxed crystals, which was experimentally verified. This cellular automata model was then coupled with the finite element (FE) method to establish a macro–micro cellular automata framework, known as the CAFE method. In this study, the microstructure evolution of a Hazelett continuous casting billet during solidification was simulated by establishing a heat flow coupling model [12] and combining it with the cellular automata method. The effects of heterogeneous nucleation parameters [13] and key process parameters, such as front box temperature, casting speed [14], and cooling strength, on grain size, dendritic crystal area ratio, and grain orientation in the solidification structure of the strip were elucidated. Additionally, our study delved into how process and nucleation parameters influence the slab’s solidification microstructure.

## 2. Establishing the Calculation Model

### 2.1. Establishing the CAFE Heat Transfer and Micro-Kinetic Model

According to the Navier–Stokes equation in fluid mechanics [15], the solidification model in this study employs the enthalpy method to address the latent heat of solidification and employs the three-dimensional transient flow and heat transfer equation, which is grounded in the conservation of mass, energy, and momentum [16], to accurately describe the solidification dynamics. The solidification of continuous casting encompasses the nucleation and growth processes of grains, each delineated by the nucleation model and growth model of micro-dynamics, respectively. The heterogeneous nucleation model [17] is utilized to depict the change in grain density during the solidification of an aluminum plate.

(1) $$\frac{dn}{d(\Delta T)}=\frac{n_{max}}{\sqrt{2\Pi }\Delta T_{\sigma }}\exp[-\frac{1}{2}(\frac{\Delta T-\Delta T_{max}}{\Delta T_{\sigma }})]$$

In the formula, dn represents the change in grain density; $n_{max}$ represents the maximum nucleation density, m^−3^; ΔT represents undercooling, K; $\Delta T_{max}$ represents the average nucleation undercooling, K; and $\Delta T_{\sigma }$ represents standard the deviation of nucleation undercooling, K.

Solidification theory indicates that grain growth is influenced by both kinetic undercooling and component undercooling. The KGT (Kurz–Giovannola–Trivedi) dendrite tip growth model [18] is able to determine the relationship between grain growth rate and undercooling. The dendrite tip undercooling typically consists of the following components:

(2) $$\Delta T=\Delta T_{c}+\Delta T_{t}+\Delta T_{k}+\Delta T_{r}$$

In the formula, $\Delta T_{c},\Delta T_{t},\Delta T_{K},\Delta T_{r}$ represent composition undercooling, thermal undercooling, kinetic undercooling, and curvature undercooling, respectively. In the solidification process of aluminum alloy, except for composition undercooling $\Delta T_{c}$, the impact of the three undercoolings, $\Delta T_{t},\Delta T_{K}$, $\Delta T_{r}$, on the solidification structure is minimal, even negligible, thus allowing the total undercooling to be simplified to $\Delta T=\Delta T_{c}$.

(3) $$\Delta T_{c}=mc_{0}[1-\frac{1}{1-\Omega (1-k)}]$$

(4) $$\Omega =Iv(\mathrm{Pe})=\mathrm{Pe}\cdot \exp(\mathrm{Pe})\cdot \int_{Pe}^{\infty }\frac{\exp(-Z)}{Z}dZ$$

(5) $$R=2\pi \sqrt{\frac{\Gamma }{mG_{c}\xi_{c}-G}}$$

(6) Pe=Rv/2D

In the formula, $c_{0}$ represents the mass fraction of solute in the liquid phase outside the spherical diffusion layer; *R* represents the radius of the dendrite tip; Ω represents solute supersaturation; Γ represents the Gibbs–Thomson coefficient; *Pe* represents the Péclet number of solutes; *Iv*(*Pe*) represents the Ivantsov function of the Péclet number; *G* represents the concentration gradient; and *m* represents the slope of the liquidus line. $\xi_{c\;}$ represents the function of the Peclet number. *D* represents the diffusion coefficient of the solute in liquid;${\;G}_{c}$ represents the concentration gradient of solute at the front edge of the dendrite tip; and v represents the growth rate of the dendrite tip. Studies generally find that there is no clear functional relationship between the growth rate of dendrite tip v and ΔT. Furthermore, to expedite computation, the actual simulation process involves appropriate modifications and fitting of the KGT model, enabling the determination of the relationship between the growth rate of dendrite tip and the aforementioned factors:

(7) $$v\left(\Delta T\right)=a_{2}\Delta T^{2}+a_{3}\Delta T^{3}$$

In the formula, a_2_ and a_3_ are the coefficients of the fitting polynomial; this is the total undercooling of the dendrite tip [19].

### 2.2. Coupled CA and FE Calculation Model

**Figure 1:**
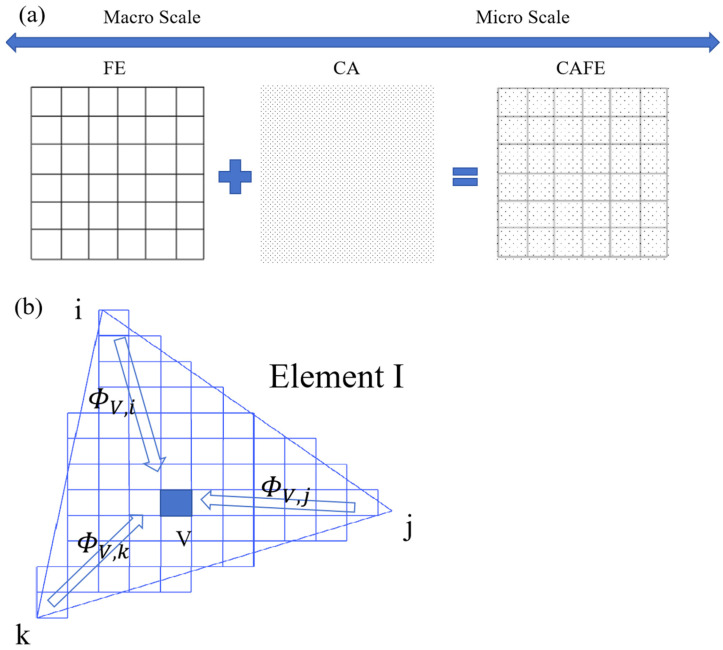
CAFE coupling model and node relationship between CA and FE. (**a**) coupled model of CAFE, (**b**) relations of CA and FE.

The finite element method (FE) partitions the solution domain into numerous interconnected subdomains, formulates an appropriate approximate solution for each, and synthesizes these to achieve the comprehensive solution desired. The cellular automata method (CA) represents a dynamic system characterized by discrete time, space, and states. Each cell operates independently, defined by time steps at specific intervals [20]. Combining the cellular automata method (CA), the macroscopic scale is reflected as the microscopic scale. As shown in Figure 1a, the finite element mesh is represented by quadrilaterals. Utilizing the FE method facilitates macro-scale simulation of the temperature field during solidification. To facilitate the coupling of the FE method and CA method within the mathematical model [16], the relationship between the CA and FE nodes is illustrated in Figure 1b. Non-zero difference factors, $\varphi_{vi}$, $\varphi_{vj}$, $\varphi_{vk}\;$, exist between the CA cell center and finite element nodes *i*, *j*, and *k* within the finite element mesh. The temperature of a CA cell is derived from FE node temperatures using an interpolation factor. Using the same interpolation factor, temperatures at each node during the solidification nucleation and growth process can be updated in real time.

## 3. Constructing a Finite Element Model and Selecting Parameters

### 3.1. Construction of Finite Element Model

**Figure 2:**
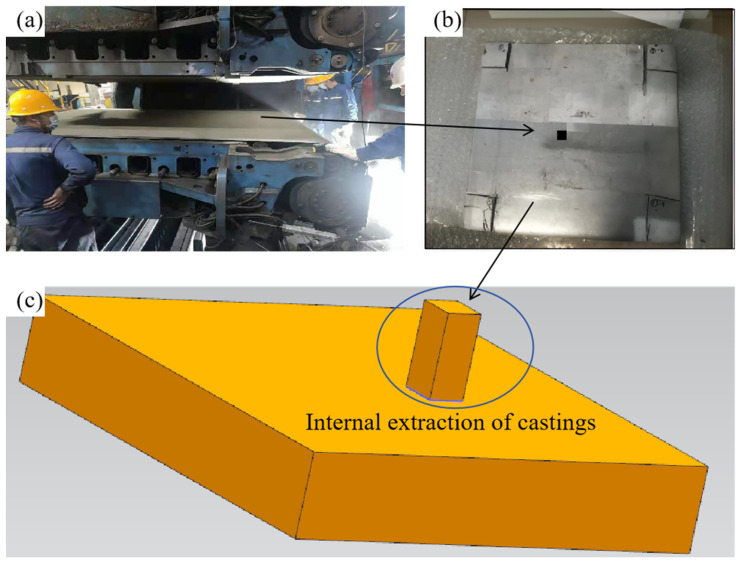
Solid and solidification zone model of casting billet, (**a**) continuous casting machine, (**b**) shear casting billet, (**c**) modeling casting billet.

**Figure 3:**
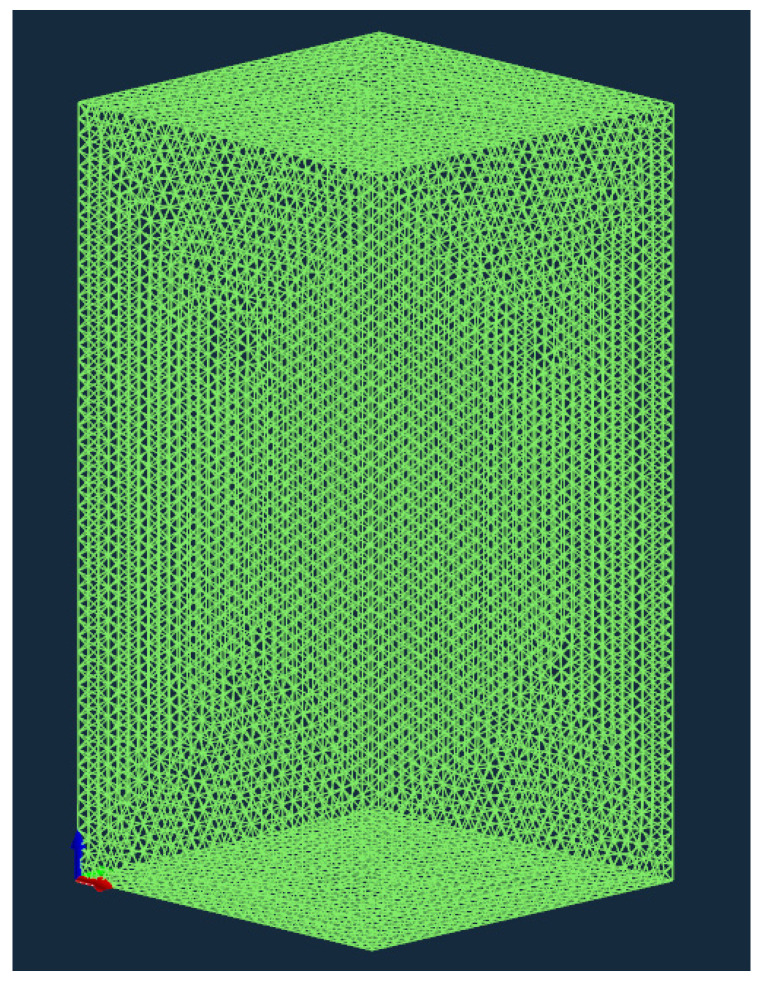
Finite element mesh model.

This study’s finite element model accurately mirrors the Hazelett continuous casting machine’s production process. To guarantee the model’s feasibility, it is possible to simplify the solidification model. Side seals are present on both sides of the Hazelett continuous casting machine, extending along the width direction of the plate. With a width-to-thickness ratio (1930/19) exceeding 100, heat transfer across the plate’s width in the Hazelett continuous casting slab is negligible. Heat transfer is disregarded in the width direction of the plate—where the continuous casting machine extends from the roller to the outlet over a distance of 2030 mm, with the slab’s width being 1930 mm and its thickness 19 mm. The continuous casting zone model was created using UG 12.0 software, with the solidification zone modeled as a primitive body. Figure 2a shows the casting machine and internal billets. Figure 2b depicts the center sampling of the 19 mm thick slab sheared at the outlet, while Figure 2c illustrates the software modeling; here, the proposed area represents the solidification zone of the primitive body, measuring 10 mm in length, 10 mm in width, and 19 mm in height. A triangular mesh divides the computational domain, with the finite element model presented in Figure 3. This article uses ProCAST 17.5 commercial software, mainly used to simulate alloy casting, which can simulate phenomena such as filling, solidification, and microstructure formation, phenomena which underpin this study. After importing the grid into the software in Figure 3, the following two finite element methods are mainly applied to simulate the solidification structure and casting process: (1) volume finite element method (VFEM), which divides the volume of castings into finite elements to simulate the solidification process; and (2) surface finite element method (SFEM), mainly used to simulate the flow and heat transfer of liquid metal.

**Figure 4:**
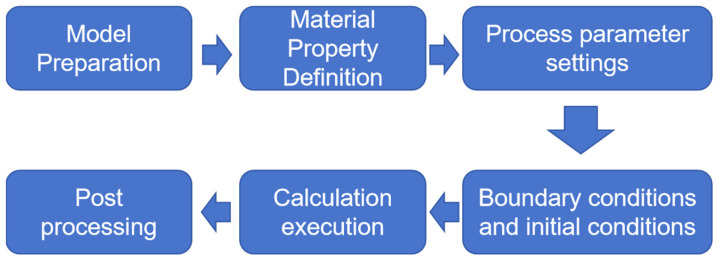
Simulation program flowchart.

Procast 17.5 software generally goes through the following steps when dealing with alloy solidification problems, as shown in Figure 4.

### 3.2. Selecting Physical Parameters and Establishing Initial and Boundary Conditions for the Solidification Model

**Figure 5:**
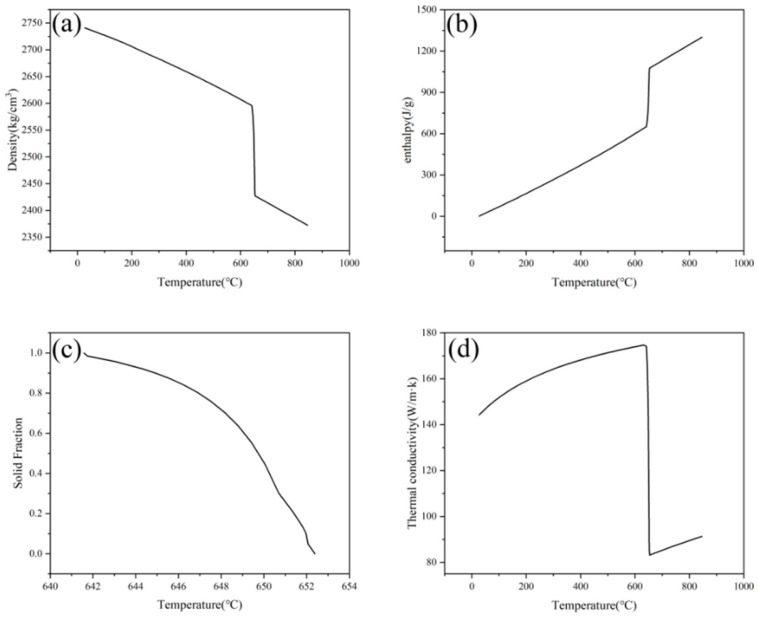
3003 aluminum alloy thermophysical parameters; (**a**) density curve, (**b**) enthalpy curve, (**c**) solid phase rate curve, (**d**) thermal conductivity curve.

The alloy chosen for the calculation model in this study is the 3003 aluminum alloy. The composition of the alloy is detailed in Table 1 [21]. Heat difference curve analysis reveals the solidus at 641 °C and the liquidus at 652 °C. JmatPro 7.0 software determines the model’s necessary thermal physical parameters, as shown in Figure 5. Based on the alloy’s composition, the CAFE simulation incorporates heterogeneous nucleation with a Gibbs–Thompson coefficient of 2 × 10^−7^ m·K. Moreover, ProCAST material library computes the dendritic growth kinetic parameters: a_2_ = 0 and a_3_ = 3.76 × 10^−5^.

**Figure 6:**
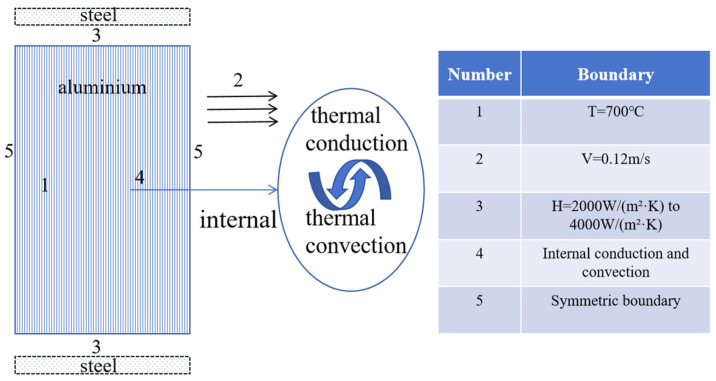
Heat transfer model and boundary conditions.

To guarantee the model’s stability, we make the following assumption during the simulation process: the air gap heat transfer layer’s thickness [22] between the low carbon steel strip and the continuous casting billet shell is assumed to be uniform. Figure 6 shows the boundary condition diagram of the heat transfer model. The initial temperature is determined by a temperature measuring gun at the continuous casting entrance. Therefore, the model assumes an initial temperature for the molten aluminum of 700 °C, a superheat of 47 °C, and a continuous casting speed of 0.12 m/s. During solidification, molten aluminum is conveyed through upper and lower layers of extremely thin low carbon steel, being cooled by circulating water inside the casting machine [23]. High-speed circulating cooling water is present behind the Hazelett low-carbon steel double strip. Referencing Pavan et al.’s research [7] on metal solidification on steel strips and incorporating the Hazelett continuous casting machine’s specialized, efficient water film cooling technology, the third boundary condition is established. Heat transfer coefficient values between the molten aluminum and the steel strip [24] range from 2000 W/(m^2^·K) to 4000 W/(m^2^·K), and the model’s surrounding boundary is symmetrical.

## 4. Determination of Nucleation Parameters and Their Effects on the Solidification Structure

### 4.1. Effect of Nucleation Density on Solidification Structure

**Figure 7:**
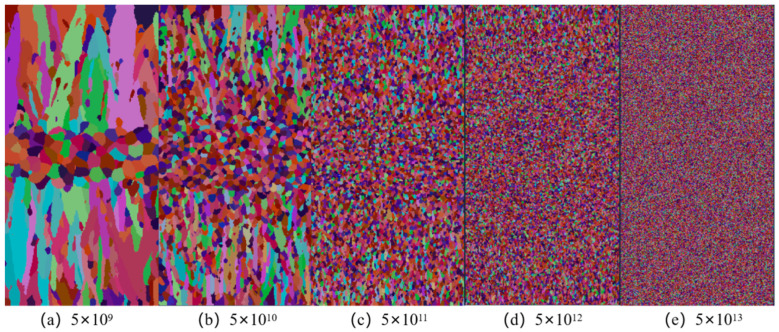
Changes in Solidification structure of casting billets with nucleation density.

For the Hazelett continuous casting simulation model, setting both body and surface nucleation parameters is necessary. Surface nucleation parameters significantly affect the casting’s surface structure during solidification, though they minimally impact the CET transformation within the casting. Conversely, body nucleation parameters critically influence the casting billet’s overall solidification structure. Therefore, surface nucleation parameters are determined at the simulation’s outset. Nucleation parameters are derived from pertinent research and a blend of experimental and computational approaches. The average supercooling for surface nucleation $\Delta T_{S,max}$ is 0.5 °C, with a standard deviation $\;\Delta T_{s,\sigma }$ of 0.1 °C, and a maximum density $N_{S,max}$ of 3 × 10^10^ m^−3^. Body nucleation parameters, reflecting the heterogeneous nucleation process, necessitate validation through experimental metallographic structures. Bulk nucleation density ranges from 10^9^ m^−3^ to 10^13^ m^−3^. The undercooling deviation $\Delta T_{V,\sigma }$ is 0.8 °C. Changing the $\Delta T_{V,max}$ and $\Delta T_{V,max}$ parameters, five nucleation densities ranging from 5 × 10^9^ m^−3^ to 5 × 10^13^ m^−3^ were chosen for simulation [2] at an undercooling of 2.5 °C. Figure 7 illustrates the slab’s solidification structure.

**Figure 8:**
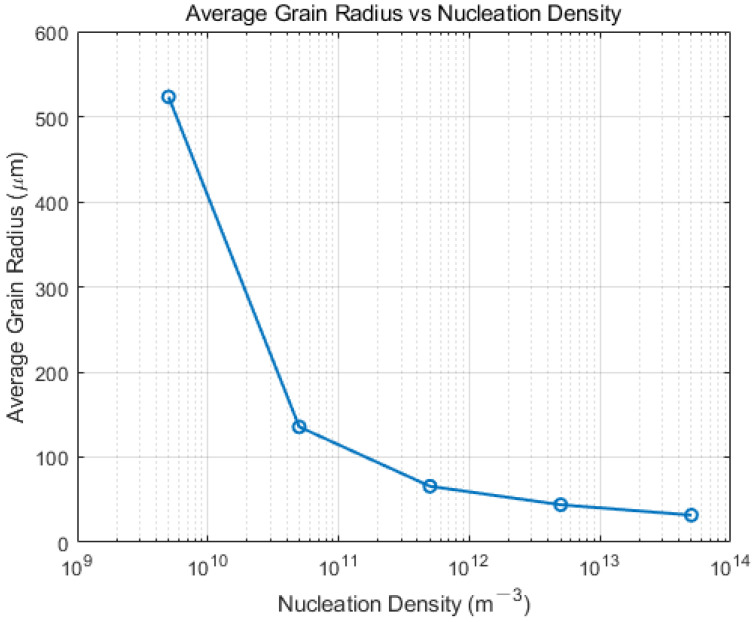
The variation in average grain radius with nucleation density.

Simulation of the continuous casting billet’s solidification process identifies nucleation density as a crucial factor influencing the microstructure. Preliminary simulation results reveal that as nucleation density increases, there is a significant rise in the proportion of equiaxed grains within the slab, accompanied by a corresponding reduction in grain size. Nucleation density escalating from 5 × 10^9^ m^−3^ to 5 × 10^13^ m^−3^ leads to an equiaxed grain proportion increase from 15.79% to 100%. Ultimately, this results in the formation of all fine equiaxed grains. Figure 8 presents a line chart correlating nucleation density with the average grain radius within the cross-sectional grain structure. It is observed that as nucleation density increases, the average radius of grains gradually decreases from 477.06 μm at a nucleation density of 5 × 10^9^ m^−3^ to 31.09 μm at a nucleation density of 5 × 10^13^ m^−3^. This phenomenon is ascribed to increased nucleation density, leading to an increase in heterogeneous nucleation particles during the nucleation. Without altering the continuous casting process parameters, dendrites inhibit each other and vie for growth space during solidification. This competition reduces the growth space for columnar crystals, prompting an earlier transformation to equiaxed crystals, thereby decreasing grain size and increasing the number of equiaxed crystals. For precise simulation of the solidified structure in the Hazelett process, selecting various maximum nucleation densities based on the alloy and casting process conditions is essential for achieving a stable and uniformly refined grain structure.

### 4.2. Effect of Undercooling Rate on Solidification Structure

**Figure 9:**
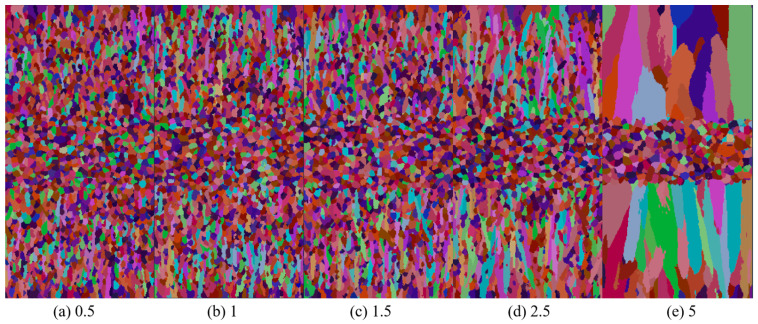
Changes in solidification structure of casting billets with undercooling rate.

To investigate the effect of undercooling on the slab’s solidification structure, alongside the aforementioned simulation results, the simulations varied nucleation undercooling while maintaining a nucleation density of 5 × 10^10^ m^−3^ and keeping other parameters constant. Figure 9 depicts the solidification structure of the slab.

**Figure 10:**
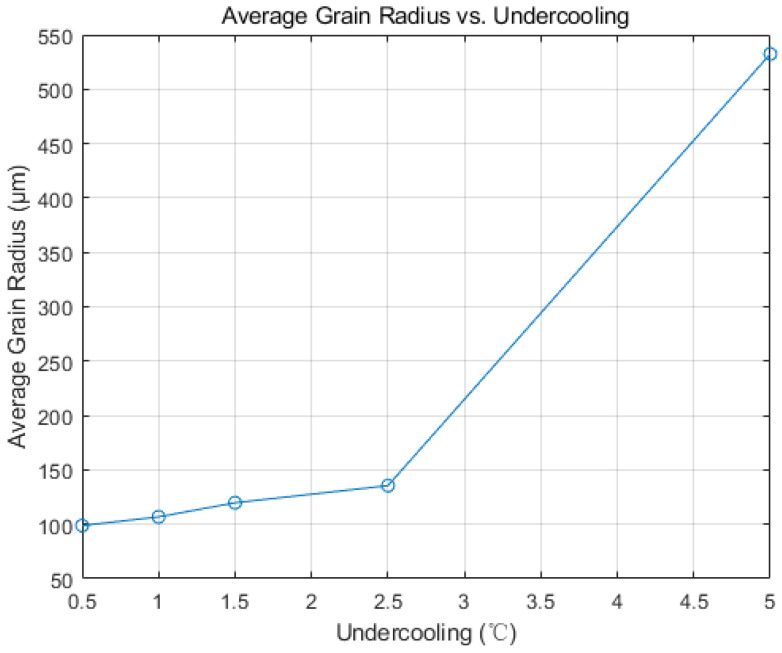
The variation in average grain radius with undercooling rate.

As undercooling intensifies, the proportion of the slab’s columnar crystal zone expands, with grains becoming increasingly coarse. This observation aligns with the Gaussian distribution of nucleation particles in the CAFE model. Figure 10 shows the average radius curves of grains under different levels of undercooling. With the increase in undercooling, the average radius of grains increases, moving from 98.81 μm at 0.5 °C to 523.19 μm at 5 °C. When the melt fails to reach the average undercooling required for nucleation, nucleation instances decrease. Upon reaching or falling below the required average undercooling, the melt rapidly undergoes extensive nucleation. Grain growth rate directly correlates with the square of the supercooling degree. With an increase in the slab’s required supercooling degree for nucleation, the number of nucleation events rises. The grain solidification front, in contact with the steel strip, experiences reduced grain hindrance, facilitating rapid growth in the direction of heat transfer. Consequently, the proportion of the slab’s columnar crystal zone increases.

### 4.3. The General Law Governing the Influence of Nucleation Parameters on Solidification Structure

**Figure 11:**
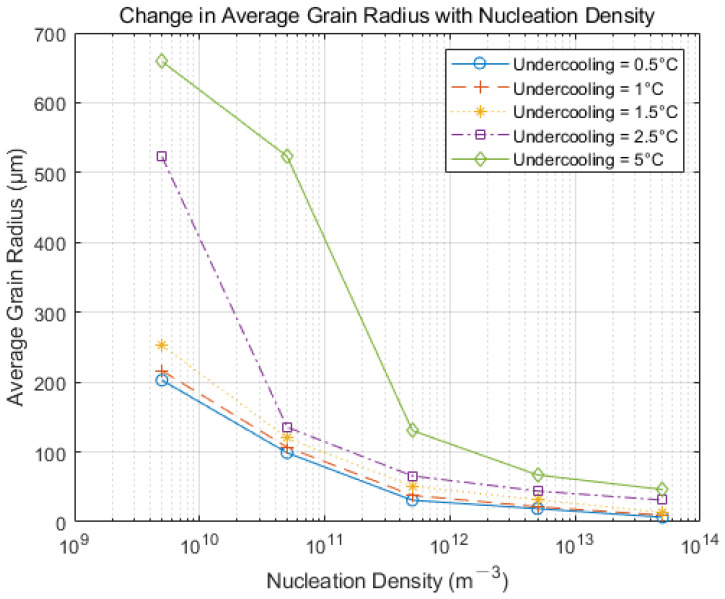
Changes in average grain radius under different undercooling degrees and nucleation densities.

Owing to the Hazelett continuous casting process’s unique characteristics, metallographic analysis shows that the cross-sectional grain structure is largely equiaxed. Therefore, extremely high nucleation density and significantly low nucleation undercooling generally diverge from the actual production results, which use the slab grain size as a reference point. For optimizing nucleation parameters in the Hazelett solidification process, five representative degrees of undercooling and five nucleation densities were selected for separate discussion. Figure 11 presents the average grain radius variation diagram for five nucleation densities, ranging from 5 × 10^9^ m^−3^ to 5 × 10^13^ m^−3^, under five different undercooling conditions: 0.5 °C, 1 °C, 1.5 °C, 2.5 °C, and 5 °C. It is evident that as nucleation density increases, the equiaxed nature of slab grains enhances, and the grains become more refined. Furthermore, the dendrite evolution trend within the slab remains consistent [25]. With increasing undercooling, the nucleation rate decreases, leading to larger overall grain sizes.

## 5. Analyzing and Verifying CAFE Simulations

### 5.1. Experimental Verification

**Figure 12:**
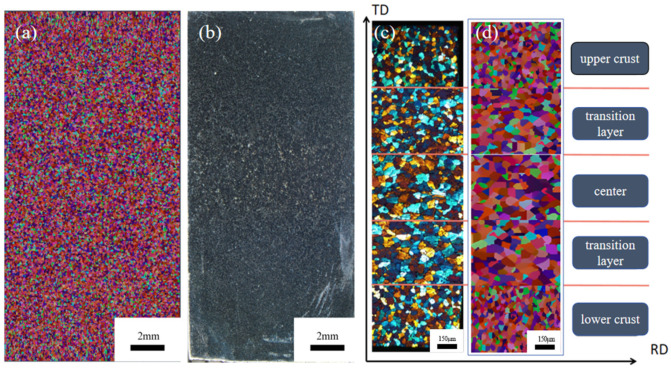
Metallographic structure diagram of RD-TD direction in casting billet and grain distribution diagram at 100× magnification. (**a**) Simulated organization, (**b**) metallographic organization, (**c**) metallographic organization of five regions, (**d**) simulated organization of five regions.

A 19 mm thick slab of 3003 aluminum alloy, produced with identical parameters to the simulation, was sampled from the Hazelett machine. Metallographic analysis was conducted on the RD-TD surface (relative to plate movement). Figure 12a depicts the simulated solidification structure of the RD-TD section of the continuous casting billet, while Figure 12b shows the overall grain distribution of the continuous casting billet, magnified 4× under a Nikon SMZ25 stereo microscope. Using Image J 1.8.0 software analysis, the average grain radius was determined to be 57.36 μm, with all grains exhibiting an equiaxed crystal morphology. Comparison with previous simulation results reveals that a nucleation density ranging from 10^11^ m^−3^ to 10^13^ m^−3^ yields a grain size and morphology distribution closely mirroring actual outcomes.

Comparing the simulated and actual grain distribution confirms the CAFE simulation’s accuracy in grain morphology and average radius. This demonstrates that the solidification structure model accurately reflects actual production and can be utilized to predict the solidification structure of Hazelett 3003 aluminum alloy. Five sections of the 19 mm casting were chosen, mechanically polished, and then anodized [26] with 10% HBF4 and 90% H_2_O solution. Under 100× magnification with a metallographic microscope, the microstructure was photographed, and the CAFE-simulated grain structure was partially enlarged. Figure 12c,d illustrates grain distribution across five regions and from the simulation, respectively.

The comparison shows compositional undercooling increases crystal nuclei numbers early in solidification. The abundant number limits their growth. Simultaneously, the chilling effect [27] accelerates the cooling rate, increases the grain tip curvature, and elevates surface energy, promoting fine grain formation. Furthermore, the Al-Ti-B refiner induces internal crystal nucleation during aluminum liquid solidification, fostering a uniform and dense structure within the aluminum alloy and mitigating segregation [28]. Within the transition and central layers, the formation of α-Al solid solutions and the precipitation of phases like Al6 (Mn,Fe) influence the kinetics of grain growth, thereby encouraging equiaxed grain development [29]. Consistent conditions such as heat accumulation, uniform elemental distribution, prolonged solidification time, lower temperature gradients, and slower cooling rates lead to grains tending towards random orientation growth.

**Figure 13:**
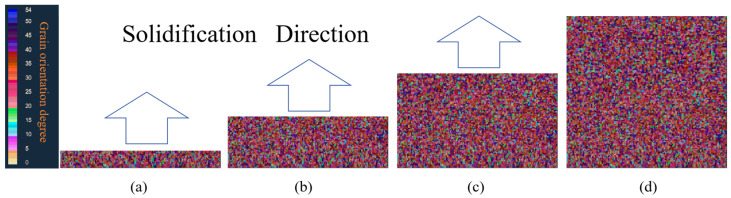
Evolution process of solidification microstructure in RD-TD section of continuous casting billet. Solidification percentage (**a**) 12.5%, (**b**) 25%, (**c**) 62.5%, (**d**) 100%.

**Figure 14:**
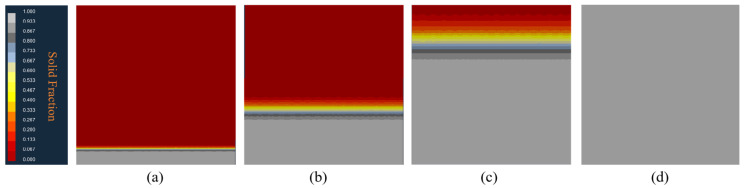
Solid fraction changes with solidification process. (**a**) 12.5%, (**b**) 25%, (**c**) 62.5%, (**d**) 100%.

Figure 13a–d depicts the solidification structure evolution process of the RD-TD section of a 3003 aluminum alloy continuous casting billet. Over time, molten aluminum cools, with solidification advancing from the exterior inward. During the initial solidification stage, the aluminum melt contacts the steel strip, with heat removed via the continuous cooling from circulating water behind the strip. A chilled crystal zone forms in the surface layer of the aluminum melt due to intense cooling, leading to significant undercooling at the edge and the formation of numerous crystal nuclei on the melt’s surface, as illustrated in Figure 13a. As solidification progresses, heat disperses outward from the slab’s core. In the Hazelett continuous casting process, the steel strip is preheated to 150 °C. Under these conditions, a minimal temperature gradient extends nucleation and solidification times, slowing heat transfer. Dendrites orient perpendicularly to the wall, enhancing supercooling. With the addition of the Al-Ti-B alloy, the grains are refined, resulting in the formation of equiaxed grains with random orientations, as shown in Figure 13b. Subsequently, a stable solidified shell of aluminum forms against the steel strip’s wall. Latent heat release during crystallization slows cooling. Free grains inhibit the growth of columnar crystals, maintaining an equiaxed crystal structure, as illustrated in Figure 13c. During the final solidification stage, the continuous casting billet’s central temperature gradient further decreases, leading to balanced temperature and heat dissipation in all directions. A minimal temperature difference hinders the formation of columnar crystals. As solidification nears completion, with reduced temperature gradients and undercooling, free grains grow at optimal undercooling and accumulate, forming an equiaxed crystal zone in the central region, as depicted in Figure 13d. Figure 14a,b shows the solid fraction changes in the RD-TD surface over time, consistent with microstructural evolution laws.

### 5.2. Effect of Process Parameters on Solidification Structure

#### 5.2.1. Effect of Initial Temperature on Solidification Structure

The pouring temperature, which is the initial temperature of continuous casting, significantly impacts the temperature field within the molten pool. Elevating the pouring temperature alters the metal’s solidification rate in the molten pool, impacting dendrite dynamics, grain size, and orientation. These elements are crucial for controlling the solidification structure.

**Figure 15:**
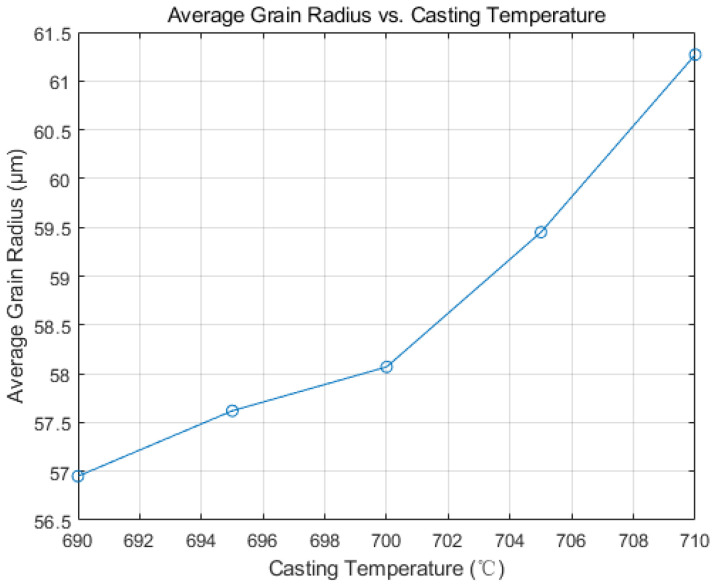
Variation in average grain radius with initial casting temperature.

Incorporating previous research and the CAFE model, this study sets the initial continuous casting temperatures to 690 °C, 695 °C, 700 °C, 705 °C, and 710 °C for simulations to investigate the impact of initial temperature on solidification structure and dendrite growth patterns. Figure 15 displays the average grain radius post-simulation. Higher initial casting temperatures lead to larger grain sizes. This occurs as the thermal gradient between the molten aluminum and the steel strip diminishes with heightened superheat, slowing the cooling rate from the slab’s edge to its center. Reduced competition among nuclei during growth allows more time for atomic diffusion, slowing the solidification front’s advance and thus extending grain growth duration. Elevating the initial temperature somewhat lessens the slab’s overall undercooling, raising the energy barrier for stable crystal nucleus formation. This leads to decreased nucleation frequency, fewer nuclei, and ultimately larger grains.

**Figure 16:**
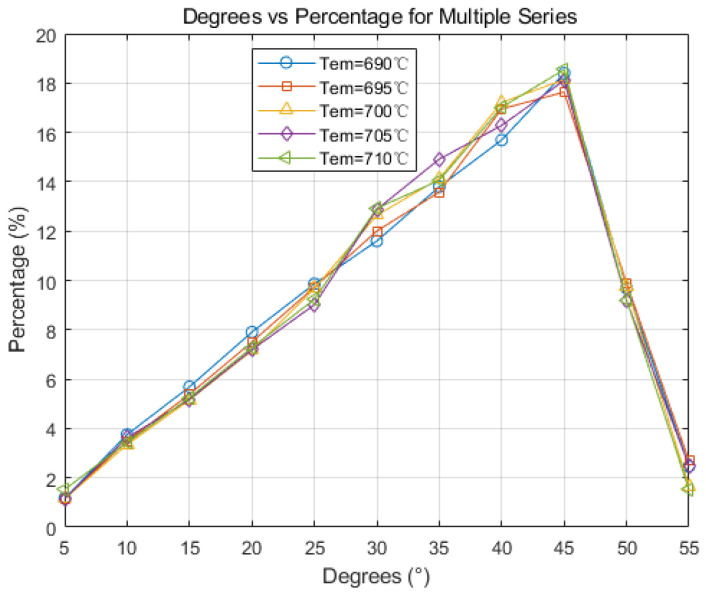
Grain orientation maps at different temperatures.

Figure 16 illustrates grain orientation distribution at different casting temperatures. The figure reveals that grain orientation follows a normal distribution at various casting temperatures, with orientations between 30° and 45° being notably prevalent. With increasing pouring temperature, high-angle grain boundaries grows, while the proportion of low-angle grain boundaries remains relatively stable. This indicates that adding high-temperature metal increases molten pool heat, restricting dendrite spacing and affecting columnar crystals. This process, occurring before rapid growth, results in the melting by the metal solution and transformation into equiaxed crystals.

#### 5.2.2. Effect of Continuous Casting on Solidification Structure

During the Hazelett continuous casting process, the steel strip and the aluminum melt move in unison within the casting machine. Cooling water is sprayed onto the back of the steel strip, forming a high-pressure water film for cooling. Varying water-cooling strengths have a direct impact on the slab’s final grain structure. For slab quality, casting speed and cooling strength require careful coordination [30].

**Figure 17:**
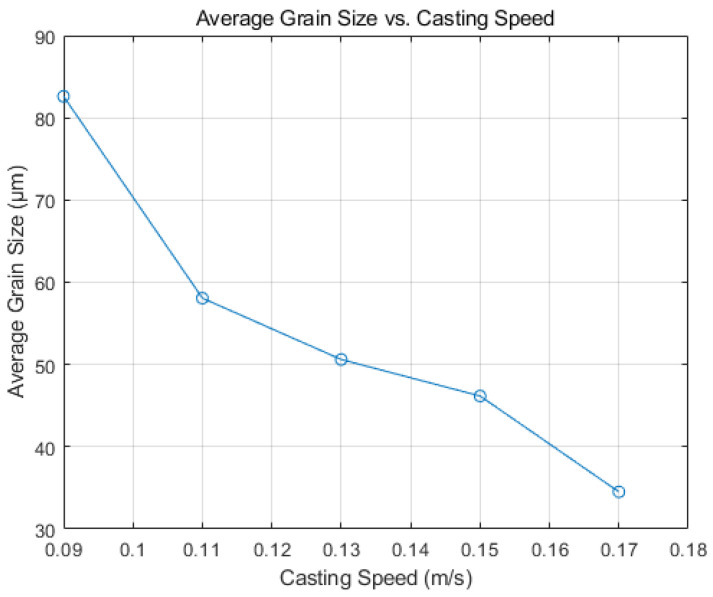
Variation in average grain radius with continuous casting speed.

In this study, the casting speeds are set to 0.09 m/s, 0.11 m/s, 0.13 m/s, 0.15 m/s, and 0.17 m/s. The simulation sets the initial temperature at 700 °C. The average grain radius resulting from the simulation is shown in Figure 17. With the increase in casting speed, the residence time of the continuous casting billet in the casting machine decreases, leading to a relative reduction in cooling intensity [31]. Consequently, the surface temperature of the casting billet rises more significantly than the central temperature. As a result, the temperature gradient at the solidification front of the casting billet diminishes, promoting the formation of a greater number of equiaxed grains. This leads to an increase in the number of grains and a decrease in grain size.

**Figure 18:**
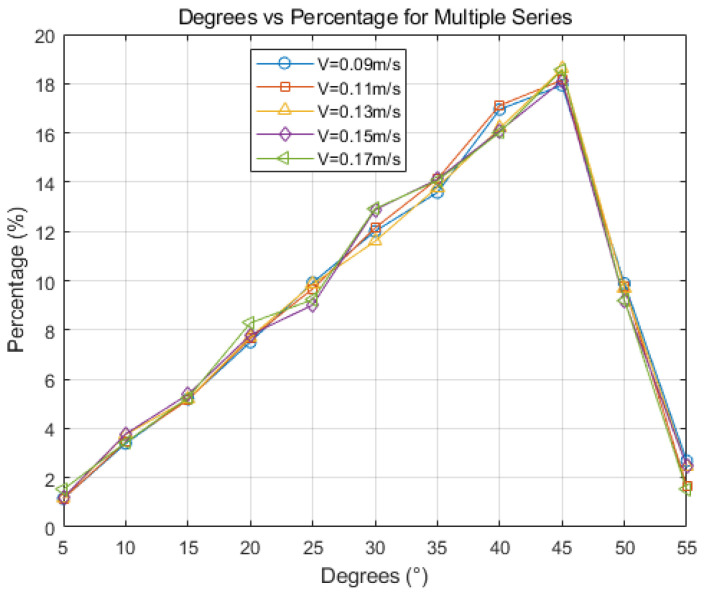
Grain orientation maps at different casting speed.

Figure 18 presents a diagram of grain orientation. With an increase in casting speed, grain orientation becomes dominated by large-angle grain boundaries, with grains between 30° and 45° being particularly prominent. Casting speed variations minimally impact grain orientation angle distribution. This is primarily because the Hazelett continuous casting machine offers precise control over cooling strength, ensuring a high compatibility between casting speed and cooling strength during the process. This has minimal impact on grain orientation. To meet grain refinement needs in the actual production process, casting speed is generally increased. At excessive casting speeds, the initial shell at the casting machine’s outlet becomes too thin, potentially leading to shell cracking and subsequent leakage or fractures [32]. Generally, controlling the casting speed to range between 0.11 m/s and 0.13 m/s is advisable.

## 6. Conclusions

(1) Based on the CAFE methodology, a comprehensive computational model for the solidification structure of the Hazelett continuous casting process was established. Simulation analysis demonstrated that adjusting the cooling rate and controlling the nucleation rate, particularly when increasing nucleation density, resulted in the slab’s grain structure manifesting a distinct equiaxed morphology, with an increase in average grain size. Observed trends were consistent across differing levels of undercooling. Metallographic analysis determined the nucleation density of the Hazelett continuous casting aluminum alloy to span between 10^11^ m^−3^ and 10^13^ m^−3^, with undercooling ranging from 1 °C to 2.5 °C. Within these nucleation parameters, the grain sizes were observed between 35 μm and 72 μm, and were primarily characterized by equiaxed grains.
(2) The initial pouring temperature significantly impacts the solidification structure of the continuous casting billet. With an increase in the initial continuous casting temperature, the grain size correspondingly enlarges. Equiaxed crystal sizes incrementally expand from 57 μm to 61.3 μm. An increase in pouring temperature leads to a higher proportion of high-angle grain boundaries, while the proportion of low-angle grain boundaries remains relatively unchanged. Higher casting temperatures diminish dendrite spacing, inhibiting the swift expansion of columnar crystals, subsequently leading to their melting and transformation into equiaxed crystals.
(3) Increasing casting speed results in a larger equiaxed grain area within the slab, and the grain size decreases accordingly, from 83 μm to 35 μm, and minimal changes in grain boundary orientation are observed. In actual production, changes in the casting speed of the continuous casting machine are harmonized with corresponding cooling conditions. The optimal range of adjustments suggests that the actual casting speed has a minimal influence on the solidification structure. The simulation results suggest that a Hazelett continuous casting speed ranging from 0.11 m/s to 0.13 m/s is optimal.

## Figures and Tables

**Table 1:** Chemical Composition of 3003 Aluminum.

Element	Si	Fe	Cu	Mn	Zn	Impurity	Al
Content	0.6	0.7	0.05~0.20	1.0~1.5	0.1	0.15	97.4~96.75

## Data Availability

Due to the fact that the data in this project contain commercially sensitive information and proprietary information of participating enterprises, according to the confidentiality agreements signed with the cooperating enterprises, the data will not be publicly available. This project is a provincial- and ministerial-level fund project jointly funded by Jiangsu University and Inner Mongolia Liansheng Group, project number 2022SYSJ0172. But in order to promote academic exchange and further research, we welcome researchers to apply for access to some data while complying with the corresponding confidentiality agreements. Interested researchers can contact the project leader via email (panhong9004@163.com). After the application, it will be reviewed based on the specific circumstances and the consent of the cooperating enterprise. Please note that all data access and usage must comply with relevant laws and regulations.
